# Who’s a Good Handler? Important Skills and Personality Profiles of Wildlife Detection Dog Handlers

**DOI:** 10.3390/ani8120222

**Published:** 2018-11-27

**Authors:** La Toya J. Jamieson, Greg S. Baxter, Peter J. Murray

**Affiliations:** School of Agriculture and Food Sciences, Wildlife Science Unit, The University of Queensland, Gatton Campus, Warrego Highway, Gatton 4343, Australia; gregbaxter36@gmail.com (G.S.B.); peter.murray@uq.edu.au (P.J.M.)

**Keywords:** dog handler, detection dog, personality, skills, dog–handler relationship

## Abstract

**Simple Summary:**

Professional working dog teams perform a range of functions faster and more accurately than other methods. Therefore, these teams are highly valuable to our society. Whilst some information is available on the skills that are important for dog handlers to possess, this isn’t always sourced from the handlers themselves. As a result, information may be missing, or the provided information may not be relevant. Through questionnaires, we collected information on the skills that wildlife detection dog handlers believe to be important for working success. Handler personality evaluations were also completed to determine whether specific personalities are better suited to this unique working field. Knowledge and understanding of dog body language and behaviour were rated highly. The handlers’ personality profiles had large ranges, indicating that no personality is attracted to, or perhaps best suited for, working with wildlife detection dogs. Dog handler dedication, training, and the dog–handler relationship are likely more influential factors.

**Abstract:**

Wildlife detection dog teams are employed internationally for environmental surveys, and their success often depends on the dog handler. Minimal research is available on the skills that dog handlers believe are important, and no research has been published on the personality profiles of wildlife detection dog handlers. This may reveal the skills that people should acquire to be successful at, or suitable for, this work. An online questionnaire was distributed to Australian and New Zealand wildlife detection dog handlers. This questionnaire provided a list of skills to be rated based on importance, and a personality assessment measured their five main personality domains (Neuroticism, Extraversion, Openness, Agreeableness, and Conscientiousness). A total of 35 questionnaires were collected, which represented over half of the estimated Australian wildlife detection dog handler population. The handlers had on average 7.2 years of dog handling experience, and 54% were female. More than half (57%) of the handlers stated that they were very emotionally attached to their dogs; however, 9% stated they were either not attached or mildly attached to their working dogs. The skill that was rated highest for importance was ‘ability to read dog body language’, and the lowest was ‘skilled in report writing’. On average, the handlers scored high in the Agreeableness domain, low in the Neuroticism domain, and average in the Extraversion, Conscientiousness, and Openness domains. However, all of the personality scores had large ranges. Therefore, a dog handler’s personality may not be as influential on their success as their training or their dog–handler bond. Further research would be beneficial regarding the direct impact that the dog–handler bond and the handler’s knowledge have on working team outcomes.

## 1. Introduction

The dog handler is potentially the most important factor affecting working dog performance [1,2,3]. The handler is responsible for more than simply following their dog in the field. They must be able to monitor and interpret their dog’s often subtle behaviours, and determine the most suitable areas and directions in which to work [4]. Underestimating the dog handler’s role can jeopardise not only the working dog’s performance, but also their welfare [1]. Whilst there is a large literature on ideal detection dog traits (reviewed by Jamieson et al. [5]), there is currently minimal information on the optimal profile of a dog handler, with handler and owner personality profiles only beginning to be researched [1,6,7,8]. Understanding what makes certain people more suited to dog handling, such as their knowledge, skills, and personality types, requires further research [8].

Wildlife detection dogs are dogs that are used for an environmental purpose, such as locating scat, plants, or live animals to determine species presence or distribution [9]. Wildlife detection dogs have been used globally for environmental surveys [10,11,12], and are particularly useful for species that occur at low densities [13,14]. Training a detection dog handler to operational standard requires significant time and dedication [14]. Therefore, it is crucial that the best candidates are selected prior to dedicating significant resources. Whilst the literature lists certain important traits for dog handlers to possess (e.g., Rebmann et al. [15], Hurt et al. [16], Minhinnick et al. [17]), there is no study that determines what wildlife detection dog handlers believe are important knowledge and skills. This is valuable information for new dog handlers or established handlers wanting to further their skills. Wildlife detection dog handlers will likely require unique skills in comparison with other detection fields due to the nature of wildlife detection work. Wildlife detection work is often required in highly remote areas, which poses multiple threats to the dog–handler team. The handler is also responsible for ensuring that their dogs pose no threat to wildlife, and may also be required to have a similar level of knowledge to wildlife ecologists to ensure survey success, which is specific to this field. Currently, there is little information published regarding the factors that influence dog handler selection [17]. Therefore, this field would benefit from an analysis of the skills and knowledge that are crucial for a detection dog team’s success [17].

When examining a person’s personality, their five main personality domains are typically measured—Neuroticism, Extraversion, Openness, Agreeableness, and Conscientiousness [18]. Previous research has determined that dog handlers have unique qualities, as shown by their five main personality domains, when compared to the personality profiles of the general population [19]. In some cases, handler personality traits have also been correlated with their dog-handling practises [8]. For example, male Polish police dog handlers were reported to have very high Conscientiousness scores, slightly above average Agreeableness and Extraversion scores, slightly below average Openness scores, and very low Neuroticism scores [19]. However, no research has determined if wildlife detection dog handlers share similar traits. As previously highlighted, wildlife detection dog handlers have unique working requirements, so determining this information may assist with dog handler selection. As a handler’s personality impacts their dog training and handling practises [8], it may also allow for dog-handler training programs to be better constructed for individuals.

This research had three main aims: (1) collect general information from Australian and New Zealand wildlife detection dog handlers to improve our understanding of their working roles; (2) determine the characteristics and knowledge that dog handlers believe are important to be successful in this specialised field; and (3) use a well-tested psychological assessment to determine if wildlife detection dog handlers share similar personality profiles. This information may assist with future wildlife detection dog handler selection and highlight skills that need to be attained to be successful in this niche field. This is especially needed in Australia, where wildlife detection dogs are still a relatively new survey method. The research hypotheses were: (1) handlers will rate the skills specific to dog handling and training highly (>4.5); and (2) handlers will have high Conscientiousness scores and low Neuroticism scores.

## 2. Materials and Methods

### 2.1. Questionnaire

We constructed a questionnaire to collect information regarding the important skills and personality profiles of wildlife detection dog handlers. The questionnaire was in three sections (see Appendix A). The first listed the traits and characteristics that the published literature has stated as being important dog handler qualities. These qualities were rated from one to five (with one being not important, and five being very important). The second section was a personality assessment, which was comprised of 120 questions that the participants used to describe themselves. The personality assessment was the International Personality Item Pool-NEO (IPIP-NEO-120; [18]). The IPIP-NEO-120 is a free, web-based personality assessment that has been demonstrated to be highly reliable with the results showing high similarity to commercial personality assessments [18,20]. The IPIP-NEO-120 assessed the handlers’ five main personality domains: Neuroticism, Extraversion, Openness, Agreeableness, and Conscientiousness [18]. The final section collected personal information such as the handlers’ age, sex, and years working as a detection dog handler.

#### Questionnaire Distribution

The questionnaire was distributed via email to dog handlers actively working with wildlife detection dogs in Australia and New Zealand. At the time of the survey, there was no formal wildlife detection dog community in Australia; therefore, it was difficult to determine the potential sample size. Based on working groups, individual dog handlers’ internet presence, and inclusion in the published literature, we estimated that there were approximately 50 individuals working within this field in Australia. New Zealand’s detection dog community is governed by the Department of Conversation (DOC). Australian wildlife detection dog teams require no formal selection or assessment. In contrast, New Zealand detection dog teams are selected by the New Zealand Department of Conservation (DOC), and teams must complete both an interim and full certificate assessment [21]. DOC estimated that there were approximately 70 dog handlers working in this field [21]. This department distributed our questionnaire to the New Zealand dog handlers. Professional dog trainers in Australia, who were also responsible for training certain handlers, were also contacted via email to distribute the questionnaire to their students (handlers). A reminder email was sent to all of the parties two weeks after initial contact in an attempt to increase sample size. All of the contacted parties were encouraged to share this questionnaire with other dog handlers who were known to them. All of the research that was completed had the University of Queensland’s Human Ethics approval (approval number: 2016001089).

### 2.2. Data Analyses

Personality profiles were calculated online using the scoring process developed by Johnson [18]. The personality profiles that were created revealed where each dog handler ranked on the scale within each personality domain. General linear models were constructed to determine the influence of the dog handler’s age, gender, country of origin, employment (professional or volunteer), experience level, or target species (native or pest) on their personality scoring or rating of skills. Pearson correlations were then measured between the dog handler’s age and years of dog handling experience, and their personality scoring. Minitab 18 was used for all of the statistical analyses. Significance was set at *p* < 0.05.

## 3. Results

### 3.1. General Handler Information

A total of 35 completed questionnaires were collected from Australian and New Zealand dog handlers. Thirty-two individual dog handlers and eight working dog organisations were sent the questionnaire. As some working dog organisations distributed these questionnaires independently, it was not possible to calculate the exact response rate. Of the returned questionnaires, 31 were from Australian handlers and four were from New Zealand. The dog handlers had a mean age of 43.9 ± 9.1 years, with 54% being female. The majority of the handlers were professionals (80%), with a mean of 7.2 years of dog handling experience. Based on their detection dogs’ target species, 57% handled native species detection dogs, 37% handled pest species detection dogs, and 6% handled both native and pest species detection dogs (Table 1).

The detection dogs used by the respondents belonged to a variety of breeds, with the majority coming from herding, gun dog, or terrier breeds (including cross-breeds). The dogs that are most commonly and currently used in Australia are Border Collies (29%), Labrador Retrievers (17%), and English Springer Spaniels (17%). Of the dog breeds listed, 26% of handlers recorded currently working with cross-breeds. Whilst the handlers varied in how emotionally attached they were to their detection dogs, all of the handlers agreed that their behaviours and stress levels impacted their dogs’ behaviours. Of the 35 handlers, 57% stated they were very emotionally attached to their dogs, while 34% were moderately emotionally attached, and 9% were mildly or not emotionally attached.

### 3.2. Important Handler Characteristics and Knowledge

The dog handlers were asked to rate a series of important dog handler skills and knowledge, which were listed in the literature. Qualities rated as most important were ‘ability to read dog body language’, ‘ability to trust in a dog’s indications’, ‘strong working ethic’, and ‘knowledgeable on dog behaviour’ (Table 2). The qualities that were rated least important were ‘skilled in report writing’, ‘strong leader’, and ‘theoretical background in ecology’.

General linear models determined no significant differences between how the handlers rated these traits based on the handlers’ age, sex, country, employment (professional or volunteer), experience level (years dog handling), or target species (native or pest species). The only exception was trait three (skilled in dog handling), which was significantly impacted by the handlers’ employment, with volunteers rating it as significantly more important (mean = 4.9) than the professional handlers (mean = 4.3; *p* = 0.011). Of the handlers who listed additional skills or qualities that they believed were important, 45% stated patience, and 35% stated experience or knowledge of their working environment.

### 3.3. Personality Profiles

The handlers’ personality domains that were the most different to the average scores were the Agreeableness and Neuroticism domains. This scoring system is based on the classifications of ‘low’, ‘average’, or ‘high’ that are provided when the personality scores were calculated online, which was developed by Johnson [18]. This online program classified a person’s results based on whether their score was in the lowest 30%, middle 40%, or highest 30% based on age and sex. For the purpose of this research, due to the differences in participants’ ages and sex, scores between 35–65 were classified as ‘average’. Scores above 65 were classified as ‘high’, and scores below 35 were classified as ‘low’. Whilst handlers’ mean personality scores differed from the average, there was a large range between the individual handlers’ scores (Table 3).

General linear models determined that the handlers’ personality domains were not affected by age, except for the Conscientiousness domain (*p* = 0.04). Age was negatively correlated to Conscientiousness (Pearson correlation = −0.348, *p* = 0.04). Similarly, the dog handlers’ personality domains were not impacted by which target species they detected (native, pest, or both), except for the Openness domain (*p* = 0.018). The dog handlers’ gender, country of residence (i.e., Australia or New Zealand), employment (professional or volunteer handler), or attachment to their dog did not impact their personality scores. The only exception was Neuroticism, which was impacted by sex (*p* = 0.049), with males having higher mean scores than females (44 and 27.3, respectively). However, there was no significant difference between the different sexes’ personality domains. Further, the dog handler’s personality domains weren’t impacted by their level of emotional attachment to their dogs.

## 4. Discussion

The returned questionnaires provided information on current wildlife detection dog handlers in Australia and New Zealand, skills they believed important, and their personality profiles. There are extensive lists of skills, knowledge, and traits that the literature states are important for detection dog handlers to possess (e.g., Rebmann et al. [15], Hurt et al. [16], Minhinnick et al. [17]). These lists aren’t always created by dog handlers. Therefore, important skills may be overlooked.

Whilst all the skills listed in the distributed questionnaire were sourced from the literature, the dog handlers clearly rated certain skills to be more important than others. The skills that were listed the highest were focused on the detection dog itself. The only traits that were directly related to the detection dogs and were rated low were knowledge of canine olfaction and experience in dog training. This was unexpected, not only because knowledge on canine olfaction has been listed as important dog handler knowledge [17,22], but also due to its relationship with environmental conditions. Dog handlers who have little experience in dog training or knowledge on dog learning theory are anticipated to struggle handling dogs in novel situations [17]. Dog handlers with dog training knowledge have been reported to be more self-confident, more aware of their dog’s working abilities, and use significantly less aversive handling methods [23]. Ensuring handlers have theoretical knowledge of dog training principles allows them to implement training practises that are efficient and humane. Therefore, future research would be beneficial to determine the performance variation between dog handlers who are also dog trainers, or have trained their current dogs, and handlers who are not experienced in dog training.

Whilst the dog handlers rated a sound knowledge of the target species relatively highly, both practical and theoretical ecological knowledge was not rated highly. This was unexpected, due to the impact that it may have on field survey success. Poor ecological knowledge may reduce the effectiveness of surveys and minimise information recorded on non-target species and habitat structure [16]. If wildlife detection dog handlers are working in close partnership with ecologists or land managers, this knowledge may not be as important. However, this knowledge is likely to aid dog handlers and potentially improve their future work [16].

There was a large variation in the handlers’ responses to how emotionally attached they were to their current detection dog/s. However, this level of attachment did not impact their personality scores. Based on the published literature, which highlights the importance and impact of the dog–handler relationship [8,24,25], it was anticipated that all of the handlers would be very emotionally attached to their dogs. This was not demonstrated in our findings. Previous research has determined that dog–handler teams with higher quality relationships also had higher performance levels and better dog–handler communication [25,26]. It could be argued that the higher the dog–handler relationship, the higher the dog’s dependence on their handler, thereby reducing the dog’s independence and performance [27]. However, a larger proportion of the literature to date has supported the hypothesis that the dog–handler relationship is positively correlated to the dogs’ problem-solving abilities and working performances [25,26,28,29,30,31]. During this project, the dog handler’s working performance was not evaluated. However, this would be beneficial in future research, with the dog teams’ performances being compared against their attachment levels. Further research is also needed on other factors that may impact dog–handler attachment and working performance, such as detection dogs’ living arrangements and conditions, daily time spent with the dog, and where the dog was sourced (e.g., adopted, purchased, or purposefully bred).

The dog handler’s personality influences the dog–handler interface and therefore the team’s performance [24]. As a result, previous research has investigated how these differences in a handler’s personality impact a working dog’s performance [8]. Within our study, there were differences between the handlers’ mean personality scores and the ‘average’ scores; however, there was a large range. This was most strongly demonstrated in the Agreeableness and Neuroticism domains. The handlers scored highly for Agreeableness. Agreeableness has previously been positively correlated with team co-operation and avoidance of conflicts, and negatively associated with dog owner-directed aggression [8,32]. Handlers scoring high for Agreeableness also use less verbal corrections, potentially creating a more positive dog–handler working relationship [8].

The dog handlers collectively scored low for the Neuroticism domain. Neuroticism in humans is related to anxious tendencies [33]. Dog handlers scoring high for Neuroticism are reported to have dogs who perform less efficiently at working tasks and take longer to respond to commands [8,33,34]. Having handlers with low Neuroticism scores may also improve a dog’s stress levels, with dogs having lower cortisol levels when handled by calm handlers [35].

Collectively, the dog handlers scored in the higher percentile of the ‘average’ score for the Conscientiousness and Extraversion domains. It has been postulated that high Conscientiousness scores will be positively associated with the dog–handler relationship and their working performance [8]. However, this association requires further research, and was not demonstrated in our results where Conscientiousness scores were not correlated with dog–handler emotional attachment. High scores for Extraversion are related to more confident individuals [36]. Whilst dog handlers should be confident in their team’s abilities, it is important that these scores don’t become too high, or else over-confidence could negatively impact their performance. Further research on the relationship between a handler’s Extraversion scores and their working performance would be beneficial. The dog handlers had an average score for the Openness domain. This was surprising, as Openness has previously been related to adaptability within a working environment [8]. However, there is currently minimal research on the relationship between a dog handler’s Openness score and their work.

## 5. Conclusions

Wildlife detection dogs and their handlers must have a compatible partnership. To achieve this, we need to begin to focus on the handlers—their selection, skills, and training—as much as we do on the dogs. Improving how we select and train detection dogs alone is not enough, nor is placing a high performing dog with an unsuitable or unknowledgeable handler going to result in good outcomes. Our research has highlighted the skills and knowledge that dog handlers believe to be important for working success. Whilst these skills were focused on wildlife detection work, certain skills are likely transferable to other detection fields. As these results weren’t always consistent with the published literature, it would be beneficial to determine how different skill sets and knowledge may impact a detection dog team’s success and performance. Further research is also required to determine the correlation between mean personality scores and dog handlers’ performance. On average, the handlers scored high in the Agreeableness domain and low in the Neuroticism domain. Assessing handlers’ personality scores may assist with highlighting individuals who have personalities suitable for this work, but this should not be the sole screening process. Whilst the mean scores of the handlers were mainly consistent with the literature, there was a very large range. Therefore, personality may not be the overall determinant of a person’s suitability for wildlife detection dog work. Rather, a potential handler’s suitability is related to how someone applies themselves to their training and work, and their dog–handler bond. Future research should begin to focus on improving how detection dog handlers are selected and trained, and ensuring that handlers are provided with the best available information to allow them to shape their own dog’s training and working plans. Optimising this dog–handler pairing will likely improve working success and efficiency [37]. Therefore, improving dog handler selection and training may improve field success and working dog welfare, which should be priorities for this highly applicable methodology.

## Figures and Tables

**Table 1:** Native and pest species, from Australia and New Zealand, listed as target species for the wildlife detection dogs. Total handlers currently handling these species-specific detection dogs are also listed.

Country	Native	Total	Pest	Total
Australia	Bell’s turtle (*Myuchelys bellii*)	1	Electric ants (*Wasmannia auropunctata*)	1
	Black-tailed antechinus (*Antechinus arktos*)	1	Feral cat (*Felis catus*)	7
	Eastern bristlebird (*Dasyornis brachypterus*)	1	Fireweed (*Chamerion angustifolium*)	1
	Emu (*Dromaius novaehollandiae*)	2	Fire ants (*Solenopsis geminate*)	1
	Koala (*Phascolarctos cinereus*)	10	Fox (*Vulpes vulpes*)	6
	Pygmy Blue-tongue lizard (*Tiliqua adelaidensis*)	1	Hawkweed (*Hieracium* spp.)	1
	Northern and Tiger quolls (*Dasyurus hallucatus* and *D. maculatus*)	8	Introduced rodents (e.g., *Rattus rattus*)	2
New Zealand	Blue duck (*Hymenolaimus malacorhynchos*)	2	Feral cat (*Felis catus*)	1
	Brown teal (*Anas chlorotis*)	1	Introduced rodents (e.g., *Rattus rattus*)	1
	Kakapo (*Strigops habroptila*)	2		
	Kea (*Nestor notabilis*)	1		
	Kiwi (*Apteryx* sp.)	1		
	South Island Takahe (*Porphyrio hochstetteri*)	1		

**Table 2:** Scoring of skills and knowledge based on their importance and relevance for wildlife detection dog handlers (1: strongly disagree it is important, 5: strongly agree it is important; SD: standard deviation).

Rank	Skill	Mean Score	SD
1	Ability to read dog body language	4.7	0.5
	Ability to trust in a dog’s indications	4.7	0.5
2	Strong work ethic	4.6	0.6
3	Knowledgeable on dog behaviour	4.5	0.6
4	Skilled in dog handling	4.4	0.8
	Ability to read wind direction	4.4	0.7
5	Navigational skills	4.3	0.8
	Sound knowledge of target species	4.3	0.8
6	High level of physical fitness/stamina	4.0	0.9
7	Practical ecological experience	3.9	1.0
8	Team player	3.7	1.1
9	Knowledgeable of canine olfactory physiology	3.6	0.9
	Experienced in dog training	3.6	1.0
10	Theoretical background in ecology	3.5	1.0
	Strong leader	3.5	1.0
11	Skilled in report writing	3.0	1.0

**Table 3:** The dog handlers’ personality scores in the five main personality domains (in bold), and the traits (shown below each domain) within these domains. The standard deviation and range is also provided.

Domain	Mean	SD	Range
**Extraversion**	**57.6**	**25.7**	**13–99**
Friendliness	58.1	27.3	7–98
Gregariousness	44.9	29.2	2–99
Assertiveness	49.4	25.8	4–95
Activity Level	71.9	18.6	36–99
Excitement seeking	51.0	25.1	12–95
Cheerfulness	56.5	22.6	5–88
**Agreeableness**	**67.5**	**24.0**	**13–99**
Trust	62.9	21.7	8–95
Morality	63.2	23.3	4–89
Altruism	55.7	25.4	13–95
Co-operation	61.3	23.9	7–89
Modesty	68.4	26.5	16–99
Sympathy	55.9	26.2	2–99
**Conscientiousness**	**57.7**	**25.3**	**2–96**
Self-efficacy	48.6	24.4	1–97
Orderliness	46.9	22.5	16–90
Dutifulness	53.3	23.1	5–95
Achievement-striving	62.3	24.8	2–91
Self-discipline	59.4	27.6	6–99
Cautiousness	60.9	24.5	18–97
**Neuroticism**	**34.9**	**27.7**	**1–82**
Anxiety	36.6	29.0	1–97
Anger	31.5	27.4	1–76
Depression	34.7	25.0	3–77
Self-consciousness	44.9	23.3	1–90
Immoderation	43.8	24.4	1–85
Vulnerability	43.1	28.7	1–99
**Openness**	**52.1**	**27.51**	**2–95**
Imagination	42.1	27.4	1–83
Artistic interests	49.7	27.0	1–89
Emotionality	41.7	30.5	1–97
Adventurousness	66.4	24.7	15–99
Intellect	47.5	28.9	1–91
Liberalism	62.0	17.5	25–89

## Appendix A

Below is the questionnaire which was distributed to Australian and New Zealand wildlife detection dog handlers.

### Appendix A.1. Important Dog-Handler Traits

(1) Please indicate which of the following qualities and characteristics you agree are important for a wildlife detection dog-handler to possess, along with the qualities you agree you personally possessed when you first began wildlife detection dog work. The ratings are classed as follows:
  **Strongly Disagree**	**Disagree**	**Neither Disagree nor Agree**	**Agree**	**Strongly Agree**
  1	2	3	4	5
  **Qualities/Characteristics of Dog-Handler**	**Important Dog-Handler Traits (Please Rate 1–5)**	**Qualities You Possessed (Please Click the Boxes)**
  High level of physical fitness/stamina		☐
  Knowledgeable of canine olfactory physiology		☐
  Skilled in dog handling		☐
  Experienced in dog training		☐
  Knowledgeable on dog behaviour		☐
  Ability to read dog body language		☐
  Theoretical background in ecology		☐
  Practical ecological experience		☐
  Sound knowledge of target species		☐
  Ability to read wind direction		☐
  Navigational skills		☐
  Team player		☐
  Strong leader		☐
  Ability to trust in a dog’s indications		☐
  Strong working ethic		☐
  Skilled in report writing		☐
(2) Do you believe there are any other important characteristics or traits for a detection dog-handler to possess?
  Personality:
  Previous experiences/knowledge:

### Appendix A.2. Personality Assessment

The following are a list of questions which are relevant to evaluating your personality type. Within the rows are statements about one’s personality, with all statements beginning with “I…”. Please indicate, by an ‘X’ in the appropriate box, how the statement describes you.

EXAMPLE ONLY	**Strongly Disagree**	**Moderately Disagree**	**Neither Agree nor Disagree**	**Moderately Agree**	**Strongly Agree**
Worry about things				X	

Please respond to the below statements with how you currently see yourself and not how you would like to see yourself. Please ensure you respond to every statement.

Personal statements	**Strongly DISAGREE**	**Moderately DISAGREE**	**Neither agree nor disagree**	**Moderately AGREE**	**Strongly AGREE**
Worry about things					
Make friends easily					
Have a vivid imagination					
Trust others					
Complete tasks successfully					
Get angry easily					
Love large parties					
Believe in the importance of art					
Use others for my own ends					
Like to tidy up					
Often feel blue					
Take charge					
Experience my emotions intensely					
Love to help others					
Keep my promises					
Find it difficult to approach others					
Am always busy					
Prefer variety to routine					
Love a good fight					
Work hard					
Go on binges					
Love excitement					
Love to read challenging material					
Believe that I am better than others					
Am always prepared					
Panic easily					
Radiate joy					
Tend to vote for socially liberal political candidates					
Sympathize with the homeless					
Jump into things without thinking					
Fear for the worst					
Feel comfortable around people					
Enjoy fantasy					
Believe that others have good intentions					
Excel in what I do					
Get irritated easily					
Talk to a lot of different people at parties					
See beauty in things that others might not notice					
Cheat to get ahead					
Often forget to put things back in their proper place					
Dislike myself					
Try to lead others					
Feel others’ emotions					
Am concerned about others					
Tell the truth					
Am afraid to draw attention to myself					
Am always on the go					
Prefer to stick with things that I know					
Yell at people					
Do more than what’s expected of me					
Rarely overindulge					
Seek adventure					
Avoid philosophical discussions					
Think highly of myself					
Carry out my plans					
Become overwhelmed by events					
Have a lot of fun					
Believe that there is no absolute right or wrong					
Feel sympathy for those who are worse off than me					
Make rash decisions					
Am afraid of many things					
Avoid contact with others					
Love to daydream					
Trust what people say					
Handle tasks smoothly					
Lose my temper					
Prefer to be alone					
Do not like poetry					
Take advantage of others					
Leave a mess in my room					
Am often down in the dumps					
Take control of things					
Rarely notice my emotions					
Am indifferent to the feelings of others					
Break rules					
Only feel comfortable with friends					
Do a lot in my spare time					
Dislike changes					
Insult people					
Do just enough work to get by					
Easily resist temptations					
Enjoy being reckless					
Have difficulty understanding abstract ideas					
Have a high opinion of myself					
Waste my time					
Feel that I’m unable to deal with things					
Love life					
Tend to vote for socially conservative political candidates					
Am not interested in other people’s problems					
Rush into things					
Get stressed out easily					
Keep others at a distance					
Like to get lost in thought					
Distrust people					
Know how to get things done					
Am not easily annoyed					
Avoid crowds					
Do not enjoy going to art museums					
Obstruct others’ plans					
Leave my belongings around					
Feel comfortable with myself					
Wait for others to lead the way					
Don’t understand people who get emotional					
Take no time for others					
Break my promises					
Am not bothered by difficult social situations					
Like to take it easy					
Am attached to conventional ways					
Get back at others					
Put little time and effort into my work					
Am able to control my cravings					
Act wild and crazy					
Am not interested in theoretical discussions					
Boast about my virtues					
Have difficulty starting tasks					
Remain calm under pressure					
Look at the bright side of life					
Believe that we should be tough on crime					
Try not to think about the needy					
Act without thinking					

### Appendix A.3. Personal Information

(a) Age:
(b) Gender: Male   Female
(c) Country of residence: Australia   New Zealand
(d) Are you a professionally employed dog-handler or a volunteer
(e) Years working as a dog-handler:
(f) What dog breed do you most frequently use for conservation detection work:
(g) What target species do you handle/train the most dogs to detect:
(h) How emotionally attached are you to the dogs you’re currently handling (please click one below):
  NOT EMOTIONALLY ATTACHED
  MILDLY EMOTIONALLY ATTACHED
  MODERATELY EMOTIONALLY ATTACHED
  VERY EMOTIONALLY ATTACHED
(i) Do you believe your stress levels influence your dog’s behaviour?   YES   NO

END OF QUESTIONNAIRE.
